# Farm-level and community aggregate economic impacts of adopting climate smart agricultural practices in three mega environments

**DOI:** 10.1371/journal.pone.0207700

**Published:** 2018-11-19

**Authors:** Le Lan, Gustavo Sain, Stanislaw Czaplicki, Nora Guerten, Kelvin Mashisia Shikuku, Godefroy Grosjean, Peter Läderach

**Affiliations:** 1 International Center for Tropical Agriculture (CIAT), Hanoi, Vietnam; 2 University of Western Australia (UWA), Perth, Australia; 3 Climate Focus, Berlin, Germany; University of Illinois, UNITED STATES

## Abstract

Recent studies highlight a growing concern over the limited adoption of climate smart agricultural (CSA) practices despite their potential benefits on adaptation, mitigation and productivity. Literature indicates several factors behind the lack of adoption including socio-demographic and economic conditions, agro-ecological scales and the nature of the practices. This paper examines to what extent and under which conditions such factors influence the adoption of CSA practices at farm, household and community level across three study sites in different continents: Vietnam, Nicaragua and Uganda. While cost benefit analysis (CBA) is employed to assess the farm-level profitability of CSA practices, the aggregate community impact disaggregated by different groups of farmer typologies with specific socio-economic features is derived from the adoption rate estimated by the relative advantage of practices and the income level of each group. Our main findings show great variation of farm-profitability of CSA practices across scales. Similar practices could generate different profitability depending on crop typologies, input access and prices, household types and local context. Regarding the aggregate profitability of CSA practices at regional scale, we found that under particular conditions, relevant factors of adoption matter to the adoption pattern and thereby affects the ranking. Such conditions include (i) high income inequality, (ii) large profitability gap of prioritized CSA practices, and (iii) large proportion of cost and benefit of the practices in the level of income. This study contributes to enhancing the prioritization process of CSA practices and provides practical guidance for research and policy to tailor the investment to appropriate end-users to assure the greatest impact for the community.

## Introduction

Urgent action is required worldwide to address climate risks to food security and to mitigate greenhouse gas (GHGs) emissions from all sectors, most prominently under the umbrella of the Paris Climate Agreement. Agriculture is at cross-road and for the first time through the Intended National Determined Contributions (INDC) more than a hundred countries pledged emission reduction from agriculture, forestry and other land uses (AFOLU)[1]. According to the most recent IPCC assessment, key food commodities such as rice, maize and wheat are projected to decrease globally in quantity [2] and quality by reducing the amount of protein and mineral concentrations [3]. These impacts threaten the most vulnerable populations, predominantly poor smallholder farmers in the developing countries. Under these anticipated complex circumstances of climate change (CC), transformation from conventional autonomous high-external-inputs dependent agriculture towards more sustainable systematic planned production is needed [4–6].

Climate Smart Agriculture (CSA) is gaining attention as a key response to CC, supporting the transformation and protection of the agricultural sector [6, 7]. CSA is defined as an approach that supports efforts from local, national and global levels to address the challenges of CC via three core components: (i) sustainably increasing productivity, (ii) contributing to adaptation and (iii) reducing GHGs emission [6, 8]. While this study mainly focuses on CSA practices and technologies at the plot and farm level, the CSA concept also encompasses approaches that strengthen the resilience of the agriculture sector by integrating climate change into the planning and implementation of agriculture strategies from farm to global policy level [6].

Agricultural practices and technologies that contribute to the achievement of the three pillars outlined above can be considered climate-smart. An example to achieve the potential CSA “triple win” is conservation agriculture which is in the following defined as the combination of minimal soil disturbance (reduced tillage or no- till), permanent soil cover (mulch), with crop rotation [9]. If the three principles of conservation agriculture are implemented, they have the potential to contribute to all the pillars of CSA by i) increasing productivity [10] ii) reducing erosion as well as increasing water storage capacity and quality and iii) enhancing soil carbon sequestration compared to conventional practices through minimum tillage practices [11].

There exists a wide range of further CSA practices which can contribute to the three aims, including for example diversification or rotation of crops, improved irrigation and residue management, land contouring and terracing as well as agroforestry [12]. Another example highlighting the potential economic benefit of CSA practices draws on an ex-ante analysis of a drought tolerant rice variety providing an estimation of increased financial benefit of US$3.3 billion in drought prone areas in South Asia [13].

Yet the adoption rate of many beneficial CSA practices is still low [6, 14]. For example, despite clear evidence over more than two decades, the proportion of total cropland under conservation agriculture in Zambia, Kenya and Zimbabwe remains lower than 1% [14]. In the last decade, a large body of research indicated that the agro-ecological, socio-demographic and economic conditions of smallholder farmers as well as technology characteristics are determinants for the adoption of CSA practices, e.g. [15–17]. However, little empirical evidence has been found on how the diversity of such factors influences the outcome of adoption of CSA practices [7, 18–20]. Another problem with CSA practices quoted in the literature is the limited information available on the economic consequences of their introduction to the farming system [14]. Given that the benefits of CSA distribute differently among scales and agro-ecologies [6], there is a particular need to understand which practices can be adopted by whom and the aggregate profits for society.

The general objective of this paper is to contribute to the current state of knowledge concerning the costs and benefits for farmers, researchers and policymakers about the introduction of new CSA practices to farming systems and their implications at the aggregate (regional) level. In order to accomplish this general objective, the paper pursues four specific objectives as follows. First, we investigate the economic profitability of a set of CSA practices for different cropping systems at farm level at different mega-environments. Second, we analyze to what extent the economic impact of CSA practices varies by farm scales and typologies in different agro-ecological zones. Third, we assess the importance of the matching of *technology characteristics–farming circumstance* in shaping the adoption potential of the technology and hence the aggregate (regional) impact of such practices. Fourth, we test the hypothesis that the technology ranked by farm profitability may differ from the ranking by aggregate economic impact.

To test our hypothesis, three countries which are highly vulnerable to CC, across three different continents were selected: Vietnam in Asia, Nicaragua in Latin America and Uganda in Africa, to capture the largest diversification possible of agro-geological, socio-economic conditions and cropping systems. In Vietnam, our focus is on annual paddy rice, Uganda on maize and beans, and in Nicaragua we concentrate on perennial cropping system of cocoa.

The paper proceeds as follows. Section 2 describes detailed information of study sites, data collection tools and methodology used for the analysis. Results are presented in section 3 while section 4 provides some discussion from the analysis. Finally, the conclusion and recommendation are followed in section 5.

## Materials and methods

### Site description

We conducted the study in three countries including Vietnam, Nicaragua and Uganda. These sites were chosen among a forty-three countries of the “Adaptation for Smallholders Agriculture Program” (ASAP)–a flagship program of the International Fund for Agricultural Development (IFAD) which aims to channel climate and environmental finance to smallholder farmers. ASAP project countries were selected based on an ex-ante estimate of the contributions of the project regarding indicators such as increased climate resilience of poor smallholder farmers, increased land area managed by climate-resilient practices, increased water use efficiency etc.

We chose the three countries in three different continents with diversified socio-economic and agro-ecological conditions to assess the weight of context-specific characteristics and their impact on adoption of CSA practices. The three study sites, compromising of the Mekong Delta Region (MDR) in Vietnam, central Nicaragua and northeastern Uganda, have significant differences in cropping systems (food crops and cash crops), geographical locations (coastal low land Asia, Central highland America and Eastern Africa) and level of development. An overview of the three study sites is provided in Table 1.

**Table 1:** Overview of three study sites Vietnam, Nicaragua and Uganda.

Characteristics	Vietnam Mekong Delta region	Nicaragua North Central	Uganda Northern region
Altitude	3–5 masl	600–1500 masl	1,078 masl
Ecological zone	Humid tropic	Humid tropic	Dryland
Climatic impact by 2099*	Increased temperature (3.1^o^-3.9°C) Sea level rise (49–105 cm)	Increased temperature (2.4^o^ – 2.8°C) Change in precipitation (-30 - +90%)	Increased temperature (3.7^o^ – 4.9°C) Change in precipitation (-20%)
No of sub-region**	5	3	4
No of sample size	170	180	453
Agriculture system	Rice-based	Cocoa	Diversified system

*Projection of climate impact under BCC-CSM-1.1 at the end of 21^st^ century (Worldbank Climate Change Climate Portal data)

**Sub-region refers to communes (Vietnam), municipalities (Nicaragua), sub-counties (Uganda) at the time of the surveys

All three sites have a relative high exposure and low level of adaptive capacity to CC [21]. In Vietnam rice production in the MDR is threatened by increased temperature and salinity intrusion; whereas cocoa in Nicaragua faces drought, soil erosion and tropical cyclones as predominant climate risks and in Uganda, changing rainfall patterns as well as frequent and prolonged droughts are affecting crop production. The range of crops are used for both consumption and commercially, which is linked to the selection of prioritized CSA practices. Cocoa is used as cash crop in Nicaragua, maize and beans are mostly used for subsistence consumption in Uganda, while rice in Vietnam is used for both purposes.

The primary site selection criteria for all sites was that households must live in regions that are affected by impacts of CC. Secondly, sub-regions, as much as possible, must be representative of the whole region, hence, various socio-economic criteria were included in sampling design including agro-ecology, poverty and ethnicity. Additional criteria were added based on the context of each country. In Vietnam, five communes of the two provinces Bến Tre and Trà Vinh in the MDR were selected. The five communes are located at different proximities to the Mekong River and the sea, hence, are situated in coastal, brackish and freshwater zones and experience different levels of soil salinity. The three municipalities Wasala, Rancho Grande and El Cuá in Nicaragua represent cocoa regions of the country with high level of CC vulnerability and are located at different altitudes to capture different climatic conditions. In Uganda, Nwoya district was selected because of its high climate variability.

### Data source

#### Included CSA practices

The CSA practices used in this study were collected from the prioritization exercise under the IFAD/CCAFS funded project “Pragmatic economic valuation of adaptation risks and responses across scales”. The CSA prioritization was developed based on the Climate Smart Agriculture Rapid Appraisal (CSA-RA) methodology [22]. The CSA-RA methodology is designed to assess the heterogeneity of local contexts and prioritize context-specific CSA options. This is a bottom-up and gender-disaggregated approach that gathers perceptions on climate vulnerability, constraints, and CSA priorities of different social groups (male & female) as well as local experts and authority levels.

The CSA-RA methodology was applied and adjusted in each study site accordingly with its characteristics and conditions. Overall, in all three countries, the prioritized CSA practices were collected through farmers’ workshops. In Vietnam, a total of five workshops was conducted involving 150 farmers. In Nicaragua and Uganda this number was 90 and 200 farmers respectively. In the workshop, farmers were asked to indicate all agricultural practices that address challenge of climate change based on their knowledge and understanding.

A list of practices generated by farmers was then revalidated and complemented with local experts’ opinions in the form of discussion in experts’ workshop. The experts were invited from Department of Agriculture and Rural Development (DARD), Department of Natural Resources and Environment (DONRE), agricultural extension department, universities, and NGOs working in climate change and agricultural related fields in the region. Having better knowledge and a broader view on the conditions of local area, these experts were asked to validate the benefit and wide-scale adoption probability of practices collected through the farmers’ workshops. Indicators of technical feasibility, applicability, profitability, sustainability, investment capital, market access and resilience to climate variability (e.g. too much rain, too hot, drought, flood, unpredictable weather, etc.) were used to prioritize practices. The context-specific prioritized CSA practices are summarized and presented in Table 2 below.

**Table 2:** Summary of prioritized CSA practices in the three case studies.

Country	CSA Practice	Problem statement	Farmer Practice (FP) and CSA practice (CSA)	Expected Impacts and Reference
**Vietnam**	**Improved variety for rice**	Higher temperature and increased salinity in soil lead to a decreased yield by 0.2%/year, leading to a current low level of 4,500kg/ha. The yield is predicted to continue decreasing over time.	**FP:** The current variety IR404 which is cropped twice a year in the two seasons but is susceptible to soil salinity. **CSA**: Change the current variety by the improved salinity tolerant variety. ‘OM6976’. However, the variety, can be planted in one season only.	➢ **Increased in yield at current level of salinity and heat:** The data from the household survey indicates that changing variety leads to an increase of 800 kg/ha (18%) per season. However, since the current variety can be grown in both season while the improved variety in only one, the annual balance favors the farmer practice: Improved variety (1 season): **5,300 kg/ha/year;** Normal variety: 4,500kg/ha/season x 2 seasons = **9,000 kg/ha/year.** ➢ **Higher price:** Price of the improved variety OM6976 in the market is 32% higher than that of the farmer variety (5000 VND/kg versus 3,800 VND/kg). *Households survey (2015)*
	**Organic fertilizer**	The loss of soil fertility have induced the use of high doses of chemical fertilizer leading to increased production costs and high level of contamination by nutrient leaching.	**FP:** Increasing doses of chemical fertilizer is used to compensate soil fertility loss. Application ratio of N-P-K (kg/ha): 100-60-60. **CSA:** Substitution of the chemical fertilizer by organic one for more sustainable and less costly rice production. Application ratio of N-P-K (kg/ha): 25-0-0. Plus 30kg Postassium, 12 kg Trichoderma and 100 kg organic fertilizer.	➢ **Yield increase:** Data from the household survey indicate that the substitution of different fertilizer leads to an increase of 587 kg/ha (12%), in the **spring season**: (from 4,750 to 5,337 kg/ha) and the same amount (11%) in the **autumn season (**from 5,300 to 5, 887 kg/ha). ➢ **Inputs costs decreases:** Reduction of using commercial chemical fertilization lead to decrease the cost of rice production. *Households survey (2015)* ➢ **GHGs emission reduces:** According to the literature [23] reducing the amount of N, P, K fertilizer added in the form of chemical fertilizer would reduce the carbon emissions by 2.8tCO2/ha.
	**Change in land uses (rice-peanuts)/crop rotation (rice-shrimp)**	Higher temperature and increased salinity in soil leads to decreased yield of the winter-spring rice season by 0.07%/year, leading to the current low yield (10,050kg/ha).The yield is predicted to continue decreasing over time leading to a need to replace the winter-spring rice.	**FP:** Rice is grown in the two seasons: Summer-autumn rice rotated with winter-spring rice**. CSA 1:** Summer-autumn rice rotated with peanuts or leguminous crops**. CSA 2:** Summer-autumn rice rotated with shrimp	➢ **Income increase:** In the case of the CSA 1, changing winter rice to peanuts reduces the production cost since less inputs are required to grow peanuts compared to winter rice. Furthermore, the price of peanuts is higher than that of rice. In the case of the CSA 2, income increases significantly due to the high price of the shrimps. *Households survey (2015)*. ➢ **Low emission:** According to a recent study [23] the emission of GHGs reduces.
	**Change in land uses (annual to perennial): Rice to coconut**	The impact of the CC endangers the viability of growing rice due to increase in temperature and salinity. Currently the rice yield has reached a low yield (9,000 kg/ha/2 seasons).	**FP:** Summer-autumn rice rotated with winter-spring rice**. CSA:** Replace rice with Coconut intercropped with sugarcane	➢ **Higher and more sustainable yield:** Coconut yield starts from year 3^rd^ and start producing 5,000 fruits/ha. Coconut yield is sustainable from year 4^th^ onward at 10,000 fruits/ha/year. *AMD secondary data (2014)*. ➢ **GHGs emission reduces:** GHGs is estimated to reduce by 14tCO2/ha. The estimation was conducted using EXACT for scenario: changing from paddy rice to >10 years perennial crop.
**Nicaragua**	**Monilia control for cocoa**	Cocoa affected by Monilia. Average losses equivalent to 235Kg/Ha	**FP:** No control**. CSA:** Manual control of Monilia control for cocoa	Monilia control may avoid up to 80% of the losses, equivalent to 188kg/ha. *Households survey (2015)*
	**Organic fertilizer**	Soil fertility and structure is deteriorating requiring more synthetic fertilization to maintain yield.	**FP:** Fertilization with synthetic fertilizers (Urea plus). Average Doses in terms of commercial product (eg. 100 Kg Urea/Ha) and of Nutrients (i.e. 46Kg/Ha N). **CSA:** Replace synthetic fertilization by organic fertilization with 266.8kg/ha. Keeping the applied number of Nutrients constants.	No expected change in yield at the beginning, but with time, soil fertility and structure will improve and yield may increase or fertilizer doses may decline.
	**Muscacea as temporal shadow in cocoa**	Cocoa crop growing without shadow could not reach its yield potential	**FP:** Cocoa is cropped without shadowing. CSA: Introduce a Muscacea with cocoa	It has been shown that shadowing the cocoa crop may increase yield by 165 racimos/ha.
**Uganda**	**Conservation agriculture**	Combined planting basins with improved maize, beans intercrop and residue retention) Frequent and severe intra-seasonal dry spells are increasingly threatening crop production in northern Uganda. The dry spell can last 2–4 weeks causing significant crop losses, food insecurity and livelihoods damages.	**FP:** local varieties of maize and beans are mono-cropped in ploughed garden. **CSA:** Drought-tolerant, early maturing, and high-yielding varieties of maize (Longe 10H) and beans (NABE 15). The two crops are intercropped under correct spacing and line planting. Planting is done in plant basins (25cm long X 15cm wide X 15cm deep). Crop residues are retained in the basins.	Plant basins will trap water when it rains. The crop residue retained will help to conserve soil moisture in the basin. The basin will avail moisture to the crops during the periods when there is moisture stress while helping to control soil erosion. The crop residues will decompose to provide soil nutrients to the crops. Overall, these expected benefits will increase productivity hence improving the livelihoods of the households. *Households survey (2015)*
	**Intercropping with drought tolerant maize and bean varieties under correct spacing and row planting**	Increased frequency of droughts, intra-seasonal variability and unpredicted rainfalls have led to difficulty in planning cropping calendars.	**FP:** Local varieties of maize and beans are mono-cropped under broadcasting plantation. **CSA:** Drought-tolerant, early maturing, and high-yielding varieties of maize (Longe 10H) and beans (NABE 15) intercropped under correct spacing and line planting.	Profits from increase in yield and reduction in quantity of seeds are higher than increasing cost associated with higher price of improved varieties. The increasing labor of row planting and measuring space is offset by the reduced labor for weeding as the beans help to suppress the growth of weeds. *Households survey (2015 and 2017)*

#### Household surveys

The International Center for Tropical Agriculture (CIAT) has collaborated with governmental partners in all three countries to get permission for conducting the surveys. The research protocols and surveys have been approved by CIAT’s Institutional Review Board (IRB).

Multiple-subject household surveys, using face to face interviews, were conducted in all three study sites. Survey questions covered a wide range of topics ranging from demographic, socio-economic data, social capital, land use and land tenure; perception of current and future climate risks and associated impact; labors and crops production activities. Data from household surveys were later used to classify the total population into homogenous groups with similar socio-economic features. Cost and benefits of CSA practices were also collected from the household surveys. The surveys in Vietnam and Nicaragua were conducted in 2015. In Uganda, the surveys were a baseline for the randomized controlled trial (RCT) designs which were performed in 2014.

In Vietnam, 170 households were randomly selected using two stage cluster procedure where a village-level sampling frame was constructed based on the number of households. At the first stage, five sub-communes were randomly selected using the Probability Proportionate to Size method [24], resulting in a higher probability of larger villages to be selected than smaller villages. As the analysis focusses on a rice-based system, 103 rice producers among 170 households were surveyed.

The sample size in Nicaragua was established considering that a minimum of 30 surveys would be necessary by farmers’ clusters–communities–to be a statistically representative sample [25]. Giving the lack of official statistics on the number of cocoa farmers in each community, the project partner NITLAPAN’s estimations of local technicians were applied. Finally, the sample size by site was established in order to get a statistical representativeness with a margin of error of 5% and a confidence level of 95%. For this, a standard statistical formula for extracting a sample from the farming population was used. Thus, from these criteria a total sample of 180 surveys were formulated.

Data from Uganda were part of a randomized controlled trial (RCT) designed to understand the role of social learning in adoption of CSA and to quantify trade-offs and synergies associated with CSA. The baseline surveys were conducted before the design of RCT which involved 1330 households which were randomly selected using multi-stage cluster sampling procedure and probability proportionate to size method. For purposes of this study, however, a sub-sample of 453 maize and beans farmers was considered.

### Methodology

#### Profitability at farming level

CBA is employed to assess the farm-level economic impact of different CSA practices. CBA refers to a systematic approach of identifying, valuing and comparing options to make decisions on whether or not to implement an investment given limited resources [26, 27]. In this study, mixed ex-ante and ex-post CBA are used depending on whether the CSA practices has been fully implemented or being planned. The incremental flow of costs and benefits of two scenarios are compared: “before implementation” and “after implementation” of the practice scenario. The incremental profitability between the two scenarios over a certain time horizon is discounted at present value, and Net Present Value (NPV) and Internal Rate of Return (IRR), the two most common indicators in CBA, are calculated using the following formula:

$${NPV}_{j}^{csa-fp}=\sum_{t=1}^{T}\frac{1}{{\left(1+r\right)}^{t}}[\sum_{j=1}^{J}p_{jt}.{\Delta Y}_{jt}^{csa-fp}-\sum_{j=1}^{J}\left({\Delta C}_{jt}^{csa-fp}\right)]$$

$$IRR=\sum_{t=1}^{T}\frac{\left({GB}_{t}-C_{t}\right)}{{\left(1+IRR\right)}^{t}}=NPV=0$$

Where *p*_*jt*_ is the price of commodity *j* in time *t*, ${\Delta Y}_{jt}^{csa-fp}$ is the annual change in yield of commodity *j* between the on-farm system with the CSA practices and in its previous state, and ${\Delta C}_{jt}^{csa-fp}$ represents the annual change in cost of using the CSA practice instead of the previous farm management system, *r* is the discount rate representing the opportunity cost of the capital. The discount rate used for analysis was chosen based on the most frequent interest rate of farmers in the area. In Vietnam, it is 9% while in Nicaragua it is 12% and in Uganda this number is 10%. The analysis period *T* is defined as the life cycle of the CSA practice which considers the time horizon since farmer adopts a CSA practice until such a time when profit of the practice starts decreasing and farmer has to re-start or implement a new one. Given that different practices involves different varieties, technologies or farming techniques, the life cycle of practices varies and thus the time horizon *T* used in the analysis also varies. The *IRR* is defined as the discount rate that makes the *NPV* equal to 0, e.g. it is the discount rate that makes the present value of the flow of future net benefits exactly equal to the initial investment. A positive *NPV* indicates positive net benefit and hence the practice should be proceeded. The same reasoning applies for the *IRR*. The higher the *IRR* is, the higher profitability of the practice can be. However, it is difficult to obtain *IRR* when there are no (high) investments in the first year.

#### Profitability at the aggregate/regional level

The aggregate ex-ante economic impact is estimated by weighting the *NPV* per unit (Ha or Farm) by the number of units adopting the CSA practice each year during the length of the planning period *T*.

To estimate the number of adopting units it is assumed that the diffusion pattern follows a logistic pattern or S curve represented by the following function:

$$Y_{t}=\frac{K}{1+e^{-a-b.t}}$$ (1)

Where *Y*_*t*_ represents the proportion of units using the CSA practice in time *t*, and *K* is the adoption ceiling which indicates the maximum proportion of the target population that will possibly adopt the CSA practice.

If data is sufficient, estimation of the logistic function may proceed by linearizing the function and estimate the parameters by linear regression or alternatively, if no data is available the function can be estimated based on the values of five parameters: i) the time when the CSA diffusion starts *t*_*o*_; ii) the initial proportion of units using the CSA; iii) The adoption ceiling *K*; iv) the actual time when the evaluation is carried over (usually the current time: *t*_1_; v) the proportion of units currently using the CSA [28].

#### Estimation of adoption ceilings

Rigorous adoption studies of agricultural technologies identify the socio-economic characteristics of populations as one of the driving factors for the adoption of an agricultural technology [29–31]. Further, the relative advantage characteristic of a new technology compared to a conventional one also shapes the adoption rate [32, 33]. In CBA studies, the probability of adoption rate is often assumed to be based on one single factor or relies on experts’ knowledge of local context in order to predict the diffusion pattern, see e.g. [34].

We entirely based this study on quantitative data/analysis and estimate the adoption ceiling *K* in three steps. In the first step, given the heterogeneity of the farm population within the recommended domain, we applied cluster analysis to group the total population into relatively homogeneous groups using the data collected through households’ surveys. Four variables of interest were included in clustering the population: i) education level of household head, ii) household labors, iii) farm size, and iv) household income. These variables were identified as key factors for adoption of a new agricultural practice or technology [33, 35]. The level of education is a relative factor for learning capacity of a CSA practice. The variable of household labors provides an understanding on labor availability for implementing a CSA practice given that some practices are labor intensive. Farm size is a proxy created to estimate the investment size of implementing a CSA practice. The income of household determines the capacity for the initial investment of CSA practices. A hierarchical cluster analysis was undertaken due to the data versatility of this method and the multiple partitions which allow selection of desired level of similarity. Since hierarchical cluster analysis is an exploratory method and does not require validity tests [36], we determine the number of clusters as three clusters. Our interest is to classify the population into three different groups according to three levels of the four included variables: low, medium and high. The Ward’s method was applied to ensure a minimum variance within each cluster and thus maximize the homogeneity of each cluster [37, 38]. To confirm that clusters are significantly different from each other, statistical tests were applied. For normally distributed variables, a multivariate analysis of variance (MANOVA) was conducted while a multivariate Kruskal-Wallis H test was used for variables that are not normally distributed. The proportion of each group compared with total sample size was later used to input in variables of adoption ceiling in the adoption model.

The second step establishes a link between the aggregate profitability of CSA practices and the value of the adoption ceiling *K*. Once the clusters have been identified, the total impact of technology *i* in terms of the present value of the net benefits (PVNB) is estimated as the sum of the *PVNB* from technology *i* over all the clusters (*j = 1*, *2*,. *J)*

$$PVNB=\sum_{j=1}^{J}{PVNB}_{j}^{i}$$

For each cluster *j* the *PVNB* for each technology *i* in year *t* is estimated as:

$${PVNB}_{j,t}^{i}={PVNB}_{ua}*Q_{j,ua,t}$$

Where *Q*_*j*,*ua*,*t*_ is the number of units of analysis (farms or units of area) in cluster *j* that adopted technology *i* in time *t*, which in turn is a function of the proportion of units of analysis that adopted technology *i* in time *t*, *Y*_*j*,*t*_ weighted by the total number of units of analysis in the cluster *Q*, the importance of the cluster.

$$Q_{j,ua,t}=Y_{j,t}*Q$$

Because *Y*_*j*,*t*_ is a function of time and the adoption ceiling *K*_*j*_ (as per Eq (1)) so it is *Q*_*j*,*ua*,*t*_ and PVNB^i^_j,t_

$${PVNB}_{j,t}^{i}=f(K_{j};Q_{j})$$

That is, the present value of the net benefits in cluster *j* accrued to the adoption of technology *i* in time *t* is mainly a function of *K* and the size of the cluster.

The third step links the value of *K* to the value of two parameters indicating the cost and profitability advantage of the practice which includes: a) size of the initial investment cost from adoption of technology *i*, relative to the farm income *RI*_*i*,*j*_ and b) size of the benefits from adoption of technology *i* relative to the farm income *RB*_*i*,*j*_

$$K_{i,j}={(RI}_{i,j};{RB}_{i,j})$$

Where,

${RI}_{i,j}=\left(\frac{I_{0,f}}{I_{f}}\right)$, the numerator representing the initial investment (Cost) at the farm level necessary to adopt the CSA practice, and the denominator the farm income.

And

${RB}_{i,j}=\left(\frac{{VPNB}_{i,f}}{I_{f}}\right)$, the numerator representing the net present value at the farm level of the flow of net benefits from adopting the CSA practice.

In order to link the empirical values of these two indicators to the value of *K* for each cluster *j*, it should be noted that both indicators are measured as proportions with values ranging from 0 to 1 and so can be linked to the Probability of Adoption in an inverse way in the case of the Cost indicator and directly in the case of the Benefit indicator:

(Probability of Adoption) _ij_ = (1 – *RI*_*i*,*j*_); in the case of the Investment indicator,

(Probability of Adoption) _ij_ = (*BI*_*ij*_); in the case of the Benefit indicator

The values of the Probability of Adoption so obtained are linked to the scale of the adoption ceiling *K* divided in quintiles, and adopted the center value of the quintile as indicated by the following Table 3:

**Table 3:** Conversion of probability of adoption into K value.

Probability of Adoption	Value of *K*
0.00–0.20	0.10
0.21–0.40	0.30
0.41–0.60	0.50
0.61–0.80	0.70
0.81–1.00	0.90

The value of the adoption ceiling *K*_*i*,*j*_ for technology *i* in cluster *j* is obtained by the simple average of the two values of *K* obtained for both indicators. This assumes that both indicators have the same importance in determining the value of the adoption ceiling.

$$K_{i,j}=\frac{\left(K_{ri}+K_{rb}\right)}{2}$$

Where *K*_*ri*_ and *K*_*rb*_ represents the values of *K* obtained for each indicator.

## Results

### Profitability of CSA practices at farm level

Table 3 briefly describes the problem which the CSA practices are aimed to solve, the Farmer Practice (the “cause”), the CSA practice (the “solution”), and the main estimated impacts of the adoption of the CSA practice, using the data from household surveys.

Based on the information shown in Table 3, CBA was performed on prioritized practices in all three countries and the results are summarized in Table 4. For rice-based systems in Vietnam, there are two out of five practices, organic fertilization and rice-peanuts rotation, where the cost of implementation is lower than that of current farming system, resulting in a negative initial investment cost, although they imply higher annual maintenance costs. The other three practices require additional cost for implementation compared to the existing farm practice, and two to five years to reach the breakeven point. Among the five practices, the rice-peanuts rotation is the most profitable.

**Table 4:** Farm-level cost benefit analysis of prioritized CSA practices in the three case studies.

Partially prioritized CSA practices	Analysis period (years)	Initial cost (US$)	NPV (US$)	IRR (%)	PP period (years)
***Vietnam***					
Rice—peanuts	10	-1252	6466	Na	Na
Rice to coconut	10	1382	3733	49	4
Rice-shrimp	10	868	3580	35	5
Improved rice variety (salinity & drought resistant)	10	19.6	3001	224	2
Organic fertilization for rice	10	-64.4	2055	Na	Na
***Nicaragua***					
Muscacea as temporal shadow in cocoa	3	35.1	181	590	1
Monila control in cocoa	6	13.3	126	88	2
Organic fertilizer for cocoa	8	73.4	60	17	8
***Uganda***					
Conservation agriculture: combined planting basins with improved maize, beans intercrop and residue retention)	15	443.2	919.7	25	6
Intercropping with drought tolerant maize and bean varieties under correct spacing and row planting	5	363.2	635.4	85	3

Note: Unit of analysis is 1 ha. Na = Not applicable

The CSA practices for cocoa in Nicaragua are diversified in time frame of analysis which depends on the life cycle of each practice. In contrast to Vietnam, all CSA practices in Nicaragua induce a cost of implementation even though they are relatively low. Among the three practices, organic fertilizer is the least profitable as its NPV is only US$ 60 and requires 8 years payback. The other two only require one or two years to recover the investment cost, however, their profitability is low, except for musacea as temporal shadow for cocoa with a significantly high rate of return (590%).

In Uganda, the profitability of conservation agriculture is significantly lower than that of intercropping with more than US$ 400 in 15 years compared to US$363 in 5 years. Given the high profit of intercropping, despite its high investment cost (more than US$600), only three years are needed to reach breakeven point. The conservation agriculture practice, however, in comparison needs six years to payback the initial investment.

### Adoption and aggregate impact

#### Clusters of population

The descriptive analysis of all clusters in three case studies are illustrated in Table 5. For all case studies, cluster 1 includes households with the lowest income and smallest farm size. For Vietnam, households in cluster 1 also have the lowest education level, highest labor density (HH labor per farm size) and are the most dependent on agriculture (46%). Cluster 2 consists of households with medium levels in all variables, while cluster 3 includes better-off households, larger farm size, better education and less dependent on agriculture.

**Table 5:** Descriptive information of clusters in the three case studies.

Cluster	Prop. of total sample (%)	Education level ^a^	HH labor ^b^ (man-day)	Farm size (ha)	Total HH income (US$)	Prop. of agriculture income (%) ^c^
*Vietnam*						
1	66	2.3 (0.8)	2.9 (1.1)	0.6 (0.4)	1644 (885)	46
2	18	3 (0.8)	3 (1.2)	0.9 (0.7)	5896 (1158)	18
3	16	3 (0.7)	3.2 (1.1)	1.1 (0.7)	15181 (5172)	14
Significance level^d^		0.02	0.009	0.001	0.000	0.006
*Nicaragua*						
1	53	3.5 (1.2)	3.9 (1.9)	7.2 (4.6)	4250 (4762)	77.5
2	36	3.7 (1)	3.1 (1.5)	7.9 (10)	4432 (4719)	20.7
3	11	3.9 (1.2)	3.3 (1.4)	8.5 (9)	6258 (10404)	32.9
Significance level		0.01	0.002	0.000	0.002	0.000
*Uganda*						
1	29.4	5.3(3.2)	0.5(0.2)	0.8(1.1)	188.1(106.8)	51
2	56.1	6.1(3.3)	0.5(0.2)	1.2(1.9)	612.5(107.9)	42.7
3	14.5	6.4(2.8)	0.5 (0.2)	2.2(2.3)	1401(202.5)	67.1
Significance level		0.000	0.000	0.000	0.000	0.000

Notes: Values presented are average across clusters and standard deviation is in the parenthesis

^a^ Education level: Vietnam: 1 = no school, 2 = completed elementary school, 3 = completed secondary school, 4 = high school, 5 = above high school; Nicaragua: 1 = can’t read or write (no school), 2 = can read or write (no school), 3 = attended elementary school, 4 = completed elementary school, 5 = attended high school, 6 = completed high school, 7 = above high school; Uganda: number of years of formal education

^b^ For Uganda this was calculated as 1 –dependency ratio

^c^ Proportion of agriculture income: proportion of rice for Vietnam; proportion of cocoa for Nicaragua; proportion of crops for Uganda

^d^ Significant at the 5% level. Kruskal-Wallis H tests was applied for variables of Vietnam and Uganda cases while Manova was applied for Nicaragua.

High inequality of income distribution is observed in the Vietnam case study. The gap in total income between group 1 and group 3 is up to $14,000 per year. However, such high income of group 3 does not originate from agricultural activities even though farmers in this group own larger farm area than the other two groups. Other variables, including the education level of household head and household labor among the three clusters, are slightly different.

In Nicaragua, income distribution is relatively similar. However, the main source of income diverges among the three clusters. The lower income cluster 1 includes households with high dependency on cocoa (77.5%) while the two other better-off clusters (2 & 3) only have small proportion of income deriving from cocoa production (20.7% & 32.9%). Nicaraguan farmers own significantly larger farms compared to those in Vietnam and Uganda. This could be a pattern of farming systems where perennial crops such as cocoa is grown. Nevertheless, if considering income per hectare, the agricultural production in central Nicaragua is not particularly profitable.

In Uganda, clusters were shaped in the form that lower income clusters also have moderately smaller farm size. Income magnitude differentiates significantly in the targeted population. Around 50% of farmers in all three clusters rely on agriculture as the main livelihood. The better-off group also has highest income dependency on agriculture which is in contrast to Vietnam and Nicaragua.

#### Adoption and aggregate impact

As indicated in the methodology, aggregate benefits of CSA practices are a function of the estimated adoption rate depending on the value of the adoption ceiling parameter “K”, which in turn is postulated to be a function of two parameters: the value of adoption investment cost relative to the farm income (I.cost/I_f_) and the value of the net benefits relative to the farm income (NPV/I_f_)

Table 6 shows the estimated values of both indicators for each cluster in the three countries.

**Table 6:** NPV and Initial investment of CSA practices versus average income of clusters.

Practices	Clusters					
	1		2		3	
	I.cost/I_f_ (%)	NPV/I_f_ (%)	I.cost/I_f_ (%)	NPV/I_f_ (%)	I.cost/I_f_ (%)	NPV/I_f_ (%)
***Vietnam***						
Rice-peanuts	-60.9	31.5	-23.4	12	-12.4	6.4
Rice to coconut	67.3	18.2	25.8	7.0	13.7	3.7
Rice-shrimp	42.2	17.4	16.2	6.7	8.6	3.5
Improved rice variety (salinity & drought resistant)	1.0	14.6	0.4	5.6	0.2	3
Organic fertilization for rice	-3.1	10	-1.2	3.8	-0.6	2
***Nicaragua***						
Monila control	0.41	4.0	0.40	3.8	0.27	2.6
Musaceas for shadowing	0.83	5.7	0.78	5.6	0.55	3.6
Organic fertilizer	0	1.8	0	1.7	0	1.2
***Uganda***						
Intercropping with drought tolerant maize and bean varieties under correct spacing and row planting	154	270	71	124	57	99
Conservation agriculture: combined planting basins with improved maize, beans intercrop and residue retention)	188	391	86	180	69	144

Following the methodology, these values were used to estimate the adoption ceiling K, and aggregate economic impact of CSA practices per clusters in the three countries. Results are described in Table 7.

**Table 7:** Projected adoption rate and aggregate impact using K value.

Practices	Initial adoption (%)	K value			Estimated diffusion (%)			Aggregate NPV ($)		
		C1^a^	C2	C3	C1	C2	C3	C1	C2	C3
***Vietnam***										
Rice-peanuts	31	0.9	0.7	0.5	89	70	46	944500	177140	49485
Rice to coconut	0	0.3	0.3	0.5	30	30	50	270018	75446	106046
Rice-shrimp	8	0.3	0.5	0.5	23	44	44	106986	74513	62748
Improved rice variety (salinity & drought resistant)	9	0.5	0.5	0.5	43	43	43	209119	58430	49205
Organic fertilization for rice	10	0.5	0.5	0.5	42	42	42	133688	37354	31456
***Nicaragua***										
Monila control	0	0.5	0.5	0.5	50	50	50	9264	14199	4193
Musaceas for shadowing	0	0.5	0.5	0.5	50	50	50	6293	9644	2848
Organic fertilizer	0	0.5	0.5	0.5	50	50	50	1923	2947	870
***Uganda***										
Intercropping with drought tolerant maize and bean varieties under correct spacing and row planting	0	0.9	0.7	0.7	72	55	55	65343	97483	25067
Conservation agriculture: combined planting basins with improved maize, beans intercrop and residue retention)	0	0.9	0.9	0.9	90	90	90	303792	579967	149134

^a^ C1: cluster 1; C2: cluster 2; C3: cluster 3

In Vietnam, the magnitude of cost and benefit of CSA practices does not affect the diffusion rate among the higher income cluster 3 as this rate remains around 40 to 50% for all five practices. In contrast, adoption rates of lower income clusters 1 and 2 are determined by level of the cost and profitability of CSA practices. For low cost and low benefit practices such as organic fertilization and improved variety, adoption rates are the same regardless of the characteristics of each cluster. The rice-peanuts practice with low cost and high benefit is expected to be adopted by the lower income groups 1 and 2. Practices which generate high cost and high benefit like shifting from rice to coconut and rice-shrimp are more affordable to the higher income clusters for implementation. Aggregate impact of CSA practices depends on the total number of analysis unit which is, in this case, the number of households in each cluster. The lower income cluster 1 represents a large percentage of the total surveyed population, thus, the profits of adopting CSA practices at community level are relatively high compared to other clusters regardless of its adoption rate. For example, 30% of households in cluster 1 adopting practice of rice-shrimp and practice of shifting rice to coconut generates higher aggregate benefits than 50% of households in cluster 3 adopting the same practices.

In Nicaragua, as almost all CSA practices for cocoa have significantly lower cost and benefit than the income level of all clusters, the diffusion rate is predicted to be 50% equally among all clusters. The aggregate impact, therefore, is higher in groups consisting of a higher number of households. By contrast, in Uganda the initial investment and benefit of CSA practices are high compared to the total income of all clusters. The conservation agriculture practice, with higher benefit and cost is expected to be adopted by 90% of all clusters despite great variation in income between those clusters. For intercropping, adoption rate is higher for lower income cluster 1 at 72% and similar for the other two clusters (55%).

### Ranking CSA practices by aggregate profitability per cluster

The ranking order of CSA practices at aggregate cluster level could be different from, or remain the same as those at farm level. Table 8 compares the ranking of profitability between 1 ha farm level and aggregate level per cluster of CSA practices in Vietnam, Nicaragua and Uganda.

**Table 8:** Comparison of ranking of CSA practices between farm profitability and aggregate profitability.

CSA practices	1 ha profitability	Aggregate profitability of practice per cluster 1	Aggregate profitability of practice per cluster 2	Aggregate profitability of practice per cluster 3
***Vietnam***				
Rice-peanuts	1^st^	1^st^	1^st^	3^rd^
Rice to coconut	2^nd^	2^nd^	2^nd^	1^st^
Rice-shrimp	3^rd^	5^th^	3^rd^	2^nd^
Improved rice variety	4^th^	3^rd^	4^th^	4^th^
Organic fertilizer	5^th^	4^th^	5^th^	5^th^
***Nicaragua***				
Muscacea as temporal shadow in cocoa	1^st^	1^st^	1^st^	1^st^
Monila control in cocoa	2^nd^	2^nd^	2^nd^	2^nd^
Organic fertilizer for cocoa	3^rd^	3^rd^	3^rd^	3^rd^
***Uganda***				
Intercropping with drought tolerant maize and bean varieties under correct spacing and row planting	1^st^	1^st^	1^st^	1^st^
Conservation agriculture: combined planting basins with improved maize, beans intercrop and residue retention)	2^nd^	2^nd^	2^nd^	2^nd^

The changes in ranking order of CSA practices are observed in cluster 1 (lowest income) and cluster 3 (highest income) for the Vietnam case study. Rice-peanuts with low cost and high return, stands at the first order of profitability, both at 1 hectare and at aggregate level of cluster 1 and 2. However, for cluster 3, rice-peanuts is positioned at 3^rd^ place behind rice to coconut (1^st^) and rice-shrimp (2^nd^) which are classified at 2^nd^ and 3^rd^ respectively for other clusters. Rice-shrimp practice brings the least benefit among all five practices for households in cluster 1 while its benefit is at 2^nd^ order for cluster 3 and 3^rd^ order for cluster 2. For case studies in Nicaragua and Uganda, there is no difference between profitability ranking at small scale area or large scale area among clusters.

## Discussion

### Farm-level profitability of CSA practices

Evidences on net economic return at farm level of CSA practices are essential inputs not only for policy makers and practitioners to develop CC adaptation strategies plans, but also for farmers to access sufficient information about the economic benefits of such practices. For policy makers, plot-level profitability supports the ranking process of various CSA practices to allocate optimal resources towards the best-bet CSA options in a given context [39]. Furthermore, there is an expected replication effect: showing a high economic return on investment of a CSA practice at an individual farm encourages the neighboring farmers to adopt it, thereby resulting in larger and longer impacts for the community [40]. For farmers, expected return on investment is one of the determinants shaping the adoption decisions of a new agricultural practice [41]. Lack of information on input costs, yields and output prices relative to conventional practices in both short-and long-term, is expected to inhibit the adoption decision of farmers [32]. Given that CSA is highly context-specific, additional studies on farm-level economic impact driven by implementing CSA practices could enrich the evidence-based CSA library to promote a better implementation of suitable CSA.

CBA, among a wide range of economic appraisal methods, is a useful approach embedded in many climate policies, adaptation and mitigation plans, see e.g. [39, 42]. CBA requires a simplistic approach in methodology and inputs uptake but can still provide a robust assessment of profitability, benefit-operation cost, benefit-investment cost, benefit-total cost and the payback period [27]. Previous studies have utilized CBA to assess profitability associated with different sets of CSA practices and climate adaptation options e.g. [43–45]. The application of CBA is specifically recommended to evaluate the economic impact of low-regret adaptation options which address the existing climate variability [46].

According to definition given by [47], all CSA practices of the three countries in this study are classified as low regret or early adaptation options. Our CBA results indicate a positive profitability for all of the prioritized CSA practices, consistent with a number of studies on cost benefit of early adaptation options in developing countries [48]. The CBA of CSA practices for rice in Vietnam produces the highest profitability amongst all, regardless of different time horizons used in the analysis. This is due to the assumption that rice production currently faces a decrease in yield over time because of salinity and drought. Thus, most of the practices for rice, in this case aims to avoid the damaging cost of current climate variability. Further, the introduced practices either decrease input costs or increase financial benefit through higher prices of new outputs and higher yields. These double counting factors lead to significant high gross benefits compared to the implementation cost. This explains unobtainable IRR in some cases (rice-peanuts, organic fertilizer and improved variety of rice) where negative incremental initial cost between the new practice and the conventional one is experienced [49]. In contrast to Vietnam, CSA practices in Nicaragua and Uganda mostly aim at improving productivity/efficiency with adaptive characteristics for future climate. The profitability of such practices is derived from a single factor: change in inputs and outputs of CSA practices keeping the outputs of conventional practices constant over time.

The extent of farm-level profitability of a CSA practice varies accordingly to crop typologies and agro-ecological contexts. In both Vietnam and Nicaragua, organic fertilizer practice is prioritized for rice and cocoa respectively. However, CBA results indicate a big difference in initial cost, profitability, and payback period between the two countries. There are two explanatory factors for this issue. Firstly, even though perennial crops are proven to uptake nitrogen from soil more efficiently, resulting in a higher yield than that of annual crops [50, 51], literature also indicates low fertilizer response in agroforestry systems [52]. In specific study sites in Nicaragua, cocoa is grown with shade trees which hinders increase in productivity. Secondly, farmers here are not interested in using chemical fertilizer to improve cocoa yield. Therefore, the difference in investment cost between the two scenarios of implementing organic fertilizer is modest. In contrast, rice in the MDR of Vietnam is used as a commercial crop for export and farmers prioritize investing in inputs for rice production. Thong, Xuan [53] reported that the proportion of fertilizer cost accounts for 40% of total cost of rice production. The use of organic fertilizer for rice in Vietnam leads to a reduction of fertilizer cost resulting in lower initial cost compared to conventional practices. Consequently, adopting organic fertilizer practice is much more beneficial for rice farmers in Vietnam than for cocoa farmers in Nicaragua. Similar debates apply for conservation agriculture and intercropping in Uganda. Although a positive net profit is a common finding between our study and a large body of literature on CA and intercropping across continents see e.g. [54–56], the level of initial cost, NPV and payback period is relatively different. For example, Daujanov, Groeneveld [54] assessing CBA of CA in Central Asia presented a payback period of more than 8 years while our case study in Africa indicated 6 years as payback period. The complex nature of CA can also shape the difference in profitability between regions. CA combines a set of sustainable practices which are applied selectively depending on site-specific conditions, and thus, the economic impact of incorporating CA alters [57].

### Diversification of adoption and community aggregate economic impact of CSA practices by agro-ecological scales and farmer typologies

Community benefit is the final outcome for policymakers or adaptation planners to determine whether or not to implement an investment. The farm-level profitability of CSA practices is therefore needed but not sufficient for policymakers to develop investment plans. The community aggregated economic impact of an agriculture practice is derived from the total number of individual farmers adopting such practice. Therefore, an ill-defined adoption would mislead the investment prioritization at macro level.

The adoption of an agricultural technology is predicted based on various factors which can be categorized into three groups: (1) farmer characteristic, (2) practice/technology characteristic and (3) characteristic of external environment (soil, climate etc.) [58]. However, the extent to which these factors influence the adoption pattern varies from case to case. For example, Knowler and Bradshaw [57] by reviewing adoption studies on conservation agriculture, shows that there is no universal convergence toward significance or insignificance of farmer’s characteristics on the adoption. This finding also applies to our case studies. In Vietnam, we found that variables related to farmer typologies as well as characteristics of CSA practices have influence on adoption rate and the aggregate community profitability. Consequently, the order of CSA practices ranked at farm-level profitability changes accordingly to different groups of the population. For example, rice-shrimp is ranked 3^rd^ in 1 ha level profitability but ranked 5^th^ in aggregated profits for the lowest income group and 2^nd^ for the highest income group. In Uganda and Nicaragua, it seems that characteristics of farmers and CSA practices has no effect on the estimated diffusion as no change is observed regarding the order of CSA practices by farm-level profitability and aggregate community profitability. If adoption rate of CSA practices is equal among different groups of population, the community profits of CSA practices will be linearly correlated with the number of households in each group.

In-depth observation of the characteristics of farmers and CSA practices indicates three possible explanatory factors for the situation: (1) magnitude of income gap, (2) magnitude of profitability gap among CSA practices and (3) size of cost and benefit of CSA practices compared to income level. First, farmers in Vietnam experience the highest inequality in income compared to the other two countries. The gap of farmers’ income in Vietnam is about $14,000 between the rich and the poor, whereas that number in Nicaragua and Uganda is around $2,000 and $1,000 respectively. This big gap leads to significant differences when comparing the size of cost and benefit of CSA practices, specifically when the magnitude of CBA among practices is also high. In Vietnam, the lowest cost CSA practice (rice-peanuts) is more than $2,000 lower than the highest cost practice (rice to coconut) while the profits derived from rice to coconut is in fact lower than rice-peanuts. Both magnitude of income gap and profitability gap of CSA practices lead to a very small proportion of poor farmers adopting the high cost CSA practice, and thereby aggregated economic impact of such practice for low income group is marginal. Hence, change in order of profitability ranking is observed. However, the high cost practices such as shifting rice to coconut and rice-shrimp are proved more resilient to increased levels of salinity [23]. This will be critical consideration for policymakers in improving the adoption rate of such practices for the low income groups.

In the case of Nicaragua, even though a gap in income among the three farmer groups is observed, the level of cost and benefit of CSA practices is too small compared to farmers’ income of all three groups, which explains no difference in the ranking results. In Uganda, there is slight differences in diffusion patterns of CSA practices observed between the lowest income group and the two better-off income groups. However, such minor differences compared to big differences in number of individual households of each group is not sufficient to drive changes in the profitability ranking.

Our study provides evidence that farmers’ characteristics determine CSA adoption level and eventually the aggregate economic impact of the adoption of a CSA practice over a certain area. Our results thereby rely on the assumption that investment in CSA is fully undertaken by farmers and depends on few characteristics. This has several implications. Adoption rate might be constrained by other factors than the implementation cost of the CSA practice, its economic benefits and its relation to farm income. For example, the lack of land ownership or of credit history will limit access to finance and adoption [59] and have not been fully taken into consideration in the clustering.

Financers are also sometimes reluctant to lend to the agriculture sector due to the perceived risks and associated transaction costs [59]. Moreover, the lack of adequate skills, limited availability of workers or inputs and climatic constraints might further reduce CSA adoption [22]. Another implication from our results is that CSA uptake might reinforce further income inequality as more affluent farmers will have better access to finance and to CSA practices and technologies that have higher positive income impacts. This situation will not only lead to a widening income gap but also an increasing resilience gap among farmers and communities. Equity considerations which have been so far underappreciated in the CSA literature [60] therefore need to be better integrated in investment planning. As a result, CSA investment plans necessitate (i) to identify which farmers will unlikely be able to have access to certain advanced CSA technologies and practices, (ii) evaluate whether such costlier practice would be potentially appropriate for these more vulnerable farmers and (iii) define strategies to unlock adoption rate. Grants from climate finance sources will therefore need to be considered.

The analysis at hand can provide a framework to help designing blended finance mechanisms and assess the degree of concessionality appropriate for various farmer groups within a CSA investment project. However, a broad range of factors will need to be carefully into consideration to ensure an optimal scaling of CSA practices [61] including improved access to information and awareness raising, technical training and capacity building [62].

## Conclusion

The objective of this paper is to assess the profitability of various CSA practices at farm-level and at aggregate community level in three different agro-ecological scales, cropping systems and socio-economic and geographic conditions. This is achieved using a mixed methodology approach. Common CBA was used to estimate farm-level profitability of CSA practices. Aggregate profitability at the regional level was generated based on adoption rate estimated using the socio-demographic and economic characteristic of farmers and the relative advantage of CSA practices. We found a convergence toward positive NPV at farm-level for all of the low-regret CSA practices at farm-level regardless of locations. However, the extent of profitability varies across study sites. In terms of aggregate profitability, our findings indicate that the magnitude of socio-economic factors, costs and benefits of CSA practices determine their weight on estimated adoption rate, and thereby aggregate profitability at regional scale. In countries with less income inequality among farmers and a small gap in profitability of CSA practices, those factors have limited influence on aggregated economic impact. However, the estimation of aggregate profitability might be strongly affected by those factors in a situation where three issues are observed: (i) large income gap in the population, (ii) when large gaps are observed in farm-level profitability among prioritized CSA practices and (iii) when cost and benefit of prioritized CSA practices are significantly high compared to income level.

From the policy perspective, understanding the profitability of CSA practices, both at small and large scale could help design investment strategies at local, national and regional levels. The empirical results show that the profitability ranking of CSA practices at small scale might be different at large scale. Depending on the local context, higher cost of CSA practices has diverse implications for different population groups. For example, the rice-shrimp rotation (high cost) brings more benefits for higher income groups than the rice-peanuts rotation (low cost). This finding is critical for policy makers to tailor appropriate investment priorities to appropriate end-users which is highlighted in various recent studies on CSA see e.g. [6, 7, 57, 63]. Furthermore, considering potential impacts of CSA practices derived from a number of adopters and integrated into the prioritization ranking, could help identify the most suitable practices and thus lead to positive and sustainable impacts for the community as a whole.
